# Supplementary material for: Identification of photoperiod-induced specific miRNAs in the adrenal glands of Sunite sheep (Ovis aries)
Source: Front Vet Sci. 2022 Jul 22;9:888207. doi: 10.3389/fvets.2022.888207 (PMC9354845; doi:10.3389/fvets.2022.888207)
Supplement: Supplementary file 1 [file Data_Sheet_1.ZIP › Supplementary file/miRDeep2 file.zip!/miRDeep2 file/Know_Structure/LP42A1/oar-mir-544.pdf]

miRBase precursor : oar-mir-544
Total read count : 328
oar-miR-544-5p read count : 308
oar-miR-544-3p read count : 17
remaining reads : 3

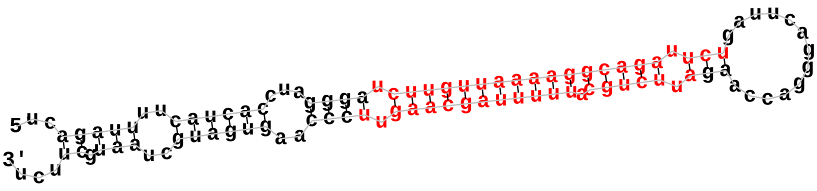


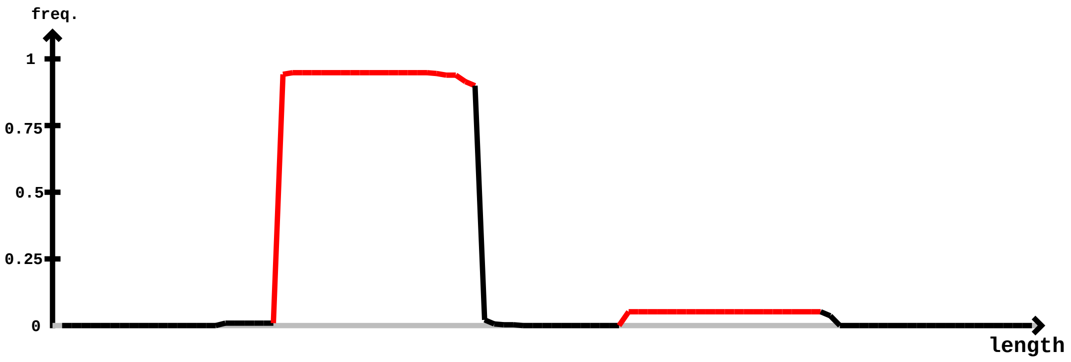


```
                                     oar-miR-544-5p
                                                                  oar-miR-544-3p
5'- ucagauuuucaucaccuagggaucuuguuaaaaggcagauucugauucagggaccaagauucugcauuuuuagcaaguucccaagugaugcuaaugcuucu  -3'  exp
    ..(((((..((((((...((((.((((((((((((((((.(((.............))).))))).))))))))))).))))..))))))..))).))...   reads   mm   sample
    ................uagggaucuuguuaaaaggcag...............................................................   1       0    seq
    ................uGgggaucuuguuaaaaggcaga..............................................................   1       1    seq
    ................uagggaucuuguuaaaaggcaga..............................................................   1       0    seq
    ......................ucuuguuaaaaggcagauu............................................................   7       0    seq
    ......................ucuuguuaaaaggcagauA............................................................   1       1    seq
    ......................ucuuguuaaaaggcagauuc...........................................................   5       0    seq
    ......................ucuuguuaGaaggcagauucu..........................................................   1       1    seq
    ......................ucuuguuaaaaggcagauucu..........................................................   267     0    seq
    ......................ucuuguuaaaaggcagauucA..........................................................   17      1    seq
    ......................ucuuAuuaaaaggcagauucu..........................................................   1       1    seq
    ......................ucuuCuuaaaaggcagauucu..........................................................   2       1    seq
    ......................ucuuguuaaaaggcagauucuU.........................................................   3       1    seq
    ......................ucuuguuaaaaggcagauucuA.........................................................   1       1    seq
    ......................ucuuguuaaaaggcagauucuga........................................................   1       0    seq
    .......................cuuguuaaaaggcagauucug.........................................................   1       0    seq
    .......................cuuguuaaaaggcagauucugauu......................................................   1       0    seq
    ..........................................................auucugcauuuuuagcaaguu......................   5       0    seq
    ..........................................................auucugcauuuuuagcaaguuU.....................   3       1    seq
    ..........................................................auucugcauuuuuagcaaguuc.....................   9       0    seq
```
